# Supplementary material for: Immunostimulatory Effect of Postbiotics Prepared from Phellinus linteus Mycelial Submerged Culture via Activation of Spleen and Peyer’s Patch in C3H/HeN Mice
Source: Pharmaceuticals (Basel). 2022 Sep 30;15(10):1215. doi: 10.3390/ph15101215 (PMC9612016; doi:10.3390/ph15101215)
Supplement: Supplementary file 1 [file pharmaceuticals-15-01215-s001.zip › pharmaceuticals-1926578-supplementary.pdf]

Table S1. Details of the ELISA kits used in the study

Antibody	Cat. No.	Manufacturer
IL-2	555148	BD Bioscience (San Diego, CA, USA)
IL-6	555240	BD Bioscience (San Diego, CA, USA)
TNF-α	88-7324-88	Invitrogen (Carlsbad, CA, USA)
IFN- γ	555138	BD Bioscience (San Diego, CA, USA)
IgG	88-50400-88	Invitrogen (Carlsbad, CA, USA)
IgA	88-50450-88	Invitrogen (Carlsbad, CA, USA)

Table S2. Analytical conditions for determining the sugar composition of PLME

HPLC system	Agilent 1120 HPLC system (Agilent Technologies, Palo Alto, CA, USA)
Stationary phase	YMC Triart C18 column (250 mm × 4.6 mm, 5 µm; YMC Co., Ltd., Kyoto, Japan) at 30°C
Mobile phase	0.1 M Phosphate buffer (pH 6.7) : Acetonitrile; 83 : 17
Equilibration time	10 min
Injection volume	20 µL
Flow rate	1 mL/min
Detector	Ultraviolet at a wavelength of 254 nm
